# Comparison of orofacial pain of patients with different stages of precancer and oral cancer

**DOI:** 10.1038/s41598-017-00370-x

**Published:** 2017-03-16

**Authors:** Yanjie Yang, Peipei Zhang, Wenlu Li

**Affiliations:** Department of Stomatology, The First Affiliated Hospital of Zhengzhou University, Zhengzhou University, Zhengzhou, Henan 450052 China

## Abstract

Orofacial pain impairs a patient's speech, swallowing, eating and interpersonal relations. Thirty-seven patients with a premalignant oral lesion, 124 patients with oral squamous cell carcinoma (OSSC), and 21 patients with a recurrence of OSSC were evaluated for their orofacial pain. The University of California San Francisco Oral Cancer Pain Questionnaire was administered to these patients at their initial visit, before they were prescribed analgesics for pain and before any treatment. Significant differences with respect to orofacial pain between the groups could be evaluatedwere observed. Patients with recurrence had the highest facial pain and patients with precancer had the lowest. Patients with OSSC and recurrence of OSSC reported significant levels of orofacial pain and functional restriction because of pain. Moreover, patients with recurrence of OSSC experienced significantly higher function-related pain, rather than pain qualities. These findings suggest that an important predictor for recurrence of OSSC may be the onset of orofacial pain that is exacerbated during function. The present study examined orofacial pain depending on the disease severity of precancer and oral cancer patients. Earlier recognition of symptoms of OSSC may improve early detection of its recurrence.

## Introduction

Oral squamous cell carcinoma (OSCC) is the sixth most common cancer in the world. Worldwide, approximately 350,000 people are diagnosed with oral cancer annually^1^. The 5-year overall survival rate of oral cancer is approximately 50%. Although modalities for the diagnosis and treatment of patients with OSCC are improving, treatment outcomes and patient prognoses have not improved greatly over the past 20 years.

For patients with advanced stage disease, radical dissection and radiation therapy (RT) are the primary treatments^2^. After survival, the primary concern of the patient with oral cancer is pain. Pain is rated as the worst symptom by patients with OSCC^3^. Although treatment can prolong survival, an adverse effect is orofacial pain, which may limit patients’ oral function and physical activity. Orofacial pain late in the treatment course may be due to surgery and microsites^4^ and may affect the patient's prognosis, as well as their daily life. Orofacial pain is reported by approximately 30–40% of patients who are treated for OSCC^5, 6^. Pain associated with the treatment of OSCC can interfere with eating, swallowing, speech, and other motor functions of the oral cavity. The aetiology of orofacial pain is multifactorial, with possible causes including RT (20–33% of cases), mucositis, epithelial atrophy, neuropathy, and temporomandibular disorder or myofascial pain. Radical excision destroys the oral structure, and resection of soft tissue and nerves may result in nociceptive nerve pain or neuropathic pain^7, 8^.

Patient survival is of the highest importance for maxillofacial surgeons, because of the low survival rate and high recurrence rate of OSCC. To ensure patient survival, drastic surgeries are often performed with the tumour section being of central importance, while aesthetics, masticatory function, and ocular motility are only of secondary importance. The drastic surgeries that are often necessary are problems that are specific to OSCC. Orofacial pain and its assessment have become increasingly important in health care, especially in the field of oral cancer screening and follow-up. Although pain is often recognized as an important symptom, a clinical aphorism has been that early head and neck cancers often go unnoticed because they are asymptomatic, and pain usually arises only when the cancer has reached a remarkable considerable size^9^.

A clinician has to manage patients with oral precancer, newly diagnosed oral cancer, and recurrence of oral cancer on a daily basis. Quite often, oral cancer is detected during regular check-ups. However, even if oral cancer is successfully treated, there is a risk that a patient will require further treatment because of recurrence. Therefore, it is very important that recurrence of OSCC be detected early. The purpose of the present study was to compare orofacial pain in groups of patients with oral precancer, newly diagnosed oral cancer, and recurrence of oral cancer. Here, we demonstrate that patients with oral cancer recurrence experience more intense pain than patients with oral precancer or newly diagnosed oral cancer.

## Results

One hundred and eighty-two patients met the eligibility criteria for the study; these patients provided written informed consent and were interviewed. Upon review of the oral biopsy specimens, 124 patients had oral cancer (squamous cell carcinoma), 37 had oral precancer (6 mild dysplasia, 4 moderate dysplasia, 24 severe dysplasia/carcinoma *in situ*, and 3 proliferative verrucous leukoplakia), and 21 had oral cancer recurrence (squamous cell carcinoma). All recurrence patients had undergone surgery and chemotherapy. The patients with precancer (N = 37) were aged between 31 and 75 years (mean 52.0 ± 10.7 years), the patients with oral cancer (N = 124) were aged between 27 and 78 years (mean 63.6 ± 14.3 years), and the patients with oral cancer recurrence (N = 21) were aged between 36 and 80 years (mean 66.2 ± 12.8 years). A comparison of the mean values of the 3 groups showed that there were a higher number of male patients in all 3 groups (Table 1).

**Table 1:** Patient characteristics.

Variables	Precancer (N = 37)	OSCC (N = 124)	Recurrence (N = 21)
Age			
<50 years	6	26	3
> = 50 years	31	98	18
Sex			
Male	28	103	17
Female	9	21	4
Occupation			
Unemployed	26	81	16
Employed	11	43	5
Marital status			
Unmarried	3	11	1
Married	34	113	20
Education level			
None	3	9	2
Elementary	5	25	7
Junior high	14	36	4
Senior high	13	43	7
College and above	2	11	1
Lifestyle habits			
Smoking	29	89	15
Alcohol	16	62	7
Betel nut	13	33	5

The majority of the patients were male (148/182, 81.3%), unemployed (123/182, 67.6%), and married (167/182, 91.8%). Most patients were educated to elementary (37/182, 20.3%), junior high (54/182, 29.7%), or senior high (64/182, 35.2%) level. In addition, 23 patients held religious beliefs. Before treatment in hospital, more than half of the patients reported betel nut use, alcohol use, and smoking before the onset of disease. Few patients reported continued betel nut use (N = 5), alcohol use (N = 7), and smoking (N = 15) after the onset of disease.

Oral cancer recurrence patients reported significantly greater functional restriction (P1 = 0.009, P2 = 0.027) and sensitivity to touch (P1 = 0.009, P2 = 0.027) in comparison with both precancer and oral cancer patients. Oral cancer recurrence patients also experienced significantly higher spontaneous sharpness (P2 = 0.000) and functional sharpness (P2 = 0.002) in comparison with oral cancer patients. In contrast, the pain levels of patients with oral precancer were not significantly different from those of patients with oral cancer in terms of either spontaneous or functional sharpness (P > 0.05). There was a significant difference in spontaneous aching (10.40 ± 10.72 vs. 28.33 ± 14.56, P = 0.001), functional aching (14.67 ± 11.91 vs. 33.33 ± 13.54, P = 0.001), and sensitivity to touch (24.67 ± 16.14 vs. 44.87 ± 21.13, P = 0.009) between the precancer patients and oral cancer patients (Table 2).

**Table 2:** Mean visual analogue scale pain scores with standard deviation for each of the 8 questions (Q) in the questionnaire for patients.

	Precancer (N = 37)	OSCC (N = 124)	Recurrence (N = 21)	P
Spontaneous intensity	26.73 ± 15.78	29.53 ± 14.80	33.53 ± 17.53	P1 = 0.693 P2 = 0.560
Functional intensity	28.47 ± 16.23	32.73 ± 16.34	37.67 ± 17.19	P1 = 0.561 P2 = 0.430
Spontaneous sharpness	10.40 ± 8.28	15.60 ± 12.85	36.40 ± 15.87	P1 = 0.278 P2 = 0.000
Functional sharpness	14.27 ± 9.29	20.87 ± 13.03	38.87 ± 16.47	P1 = 0.225 P2 = 0.002
Spontaneous aching	10.40 ± 10.72	28.33 ± 14.56	35.73 ± 13.06	P1 = 0.001 P2 = 0.129
Functional aching	14.67 ± 11.91	33.33 ± 13.54	40.93 ± 14.48	P1 = 0.001 P2 = 0.197
Sensitivity to touch	24.67 ± 16.14	44.87 ± 21.13	60.87 ± 19.72	P1 = 0.009 P2 = 0.027
Functional restriction	27.20 ± 15.52	46.20 ± 16.20	59.93 ± 10.81	P1 = 0.001 P2 = 0.014

P1 = recurrence group vs. pre-cancer group. P2 = recurrence group vs. OSSC group.

## Discussion

Pain control and weight maintenance are especially challenging in patients with oral cancer. Many oral cancer patients experience symptoms that are more severe than those produced by other cancers. They also experience difficulty with eating, drinking, swallowing, and speaking^10, 11^. In an attempt to improve management of cancer pain, the World Health Organization and the Agency for Health Care Policy recommends characterizing cancer pain as mild, moderate, or severe, and reevaluating pain levels throughout treatment. Despite this recommendation, there has not been an instrument available to quantify and characterize oral cancer pain. To address the need for such an instrument, in 2004, Connelly *et al.* published the UCSF-OCPQ to quantify patients’ pain and to identify the functions that lead to oral cancer pain^7^. Kolokythas *et al.* found the UCSF-OCPQ to be an effective tool in quantifying pain from oral cancer^12^.

In our study, oral cancer recurrence patients reported significantly greater functional restriction (P1 = 0.009, P2 = 0.027) and sensitivity to touch (P1 = 0.009, P2 = 0.027) in comparison with both precancer and oral cancer patients. Oral cancer recurrence patients also experienced significantly higher spontaneous sharpness (P2 = 0.000) and functional sharpness (P2 = 0.002) in comparison with oral cancer patients. Thus, we found that patients with oral cancer recurrence reported significant spontaneous and function-related pain at the time of cancer recurrence. These findings suggest that an important predictor of oral cancer recurrence may be the onset of orofacial pain that is exacerbated during function.

In the present study, we found that sharpness and intensity of oral pain in all 3 groups were related to function rather than being spontaneous. Patients with oral cancer report pain during oral functions, including talking, eating, and drinking. In the OSCC groups, there was a significant difference between spontaneous aching (28.33 ± 14.56) and functional aching (33.33 ± 13.54). The functional sharpness (20.87 ± 13.03) pain level was significantly higher than that of spontaneous sharpness (15.60 ± 12.85). Similarly, patients with bone metastases develop acute breakthrough pain with movement of the involved skeleton^13^.

Oral cancer pain is more likely to be a result of the sensitization and/or activation of primary nociceptive afferents by mediators liberated by the cancer and associated cells^14^. The intense, spontaneous, sharp and aching pain reported by oral cancer patients suggests the sensitization and/or activation of both Ad and C fibres in oral cancer pain^15^. Cancer and associated cells in the cancer microenvironment may release a variety of pain mediators, including adenosine triphosphate, bradykinin, cytokines, chemokines, nerve growth factor, prostaglandins, and several vascular factors, including such as endothelin 1 and vascular endothelial growth factor to either excite or sensitize nociceptive primary afferents. Arachidonic acid metabolites, such as prostaglandins, are produced by various cancers, including oral cancer, and are well-known to sensitize nociceptive primary afferents^16^.

In our study, we found that there was no correlation between tumour size and reported pain levels, suggesting that oral cancer pain is not a result of the mass effect of the tumour. This finding is consistent with the clinical observation that small oral carcinomas can be profoundly painful. We did find increased levels of pain in the OSCC patients with lymph node metastasis. The process of tissue infiltration leading to metastasis could be responsible for the increased oral pain reported in these patients. The mechanism of pain in these patients likely involves either perineural infiltration and/or nociceptor hypersensitivity. The mediators responsible for infiltration and metastasis might also be involved with nociceptor hypersensitivity^7^.

There are a number of limitations in our study. The study period included only a few patients with a relapse (recurrence). Despite the low number of recurrence patients included in this study, we were able to establish statistically relevant correlations. Further studies will be performed using a longitudinal design to determine if and how the oral cancer pain of a patient develops during progression of the disease.

In conclusion, earlier recognition of symptoms of OSSC may improve early detection of the recurrence of OSSC. Further investigations into the correlations between pain parameters and the specific biology of OSSC may improve quality of life and survival for OSSC patients.

## Methods

### Ethics statement

This study complied with the guidelines of the Declaration of Helsinki and was approved by the Medical Ethics Committee of the First Affiliated Hospital, Zhengzhou University (Zhengzhou, China). Since this study involved retrospective review of existing data, a waiver of written informed consent was obtained from the Institutional Review Board. All primary data was collected according to procedures outlined in epidemiology guidelines that strengthen the reporting of observational studies. Patient information was anonymized and de-identified prior to analysis.

## Patients

This study was previously approved by the Ethics Committee on Research of the School of Dentistry at Zhengzhou University. From August 2012 to May 2015, a cross-sectional analysis was performed on 124 OSCC patients (76 men, 48 women; mean age = 58.3 ± 8.2 years), 37 oral precancer patients (23 men, 14 women; mean age = 51.0 ± 13.2 years), and 21 patients with squamous cell carcinoma recurrence (12 men, 9 women; mean age = 61.2 ± 9.3 years) who were referred to the Department of Stomatology at the First Affiliated Hospital of Zhengzhou University. A comparison of the mean values of the 3 groups showed that they were well matched for age (P = 0.285) and sex (P = 0.059); however, there was a higher number of male patients in all 3 groups (Table 1).

### Study inclusion criteria and protocol

Only patients with an oral premalignant lesion (leukoplakia, erythroplakia, lichen planus), oral cancer, or and aoral cancer recurrence were included in this study. Potential patients would have beenPatients were excluded from the study because of foreseeable missing opportunity ofif they were unable to attend a follow-up examination, were pregnant or nursing, had undergone recent surgery, or had heart, infectious, circulation, systemic, malignant, or immune system diseases, blood coagulation disorders, or allergic reactions to pharmaceuticals and antibiotics. In addition, patients diagnosed with a psychiatric condition, or had an addiction to pain medications or recreational drugs, or had taken pain medications in the previous 6 months were also excluded.

Demographic information was also collected for each patient, which included age, sex, racial/ethnic identity, current smoking and high-risk drinking status, oral lesion location, and tumour size.

### UCSF Oral Cancer Pain Questionnaire

The University of California San Francisco Oral Cancer Pain Questionnaire (UCSF-OCPQ) was used to assess patients’ pain and to identify the functions that lead to oral cancer pain. The questionnaire was designed to differentiate function-related and spontaneous pain, as well as to determine the nature and quality of pain. The questionnaire is specifically designed for patients with oral cancer and was used because it has been shown to be better for demonstrating changes in pain that occur because of illness.

The UCSF-OCPQ was administered to patients meeting the inclusion criteria at their initial visit, and before they were prescribed analgesics for any orofacial pain or received any treatment. This questionnaire, consisting of 8 questions on a visual analogue scale of 0–100 mm, has been validated previously^12^. Briefly, the 8 questions differentiate spontaneous and function-related pain, and determine the quality of pain. Questions 1 through 6 examine the intensity, sharpness, and aching nature of orofacial pain. Question 7 focuses on the degree of sensitivity to touch. Question 8 determines the level of functional restriction as a result of orofacial pain. Patients were instructed to place a vertical line along the scale to approximate their orofacial pain level (if any).

### Statistical analysis

Statistical analysis was conducted using SPSS for Windows version 20.0 (SPSS Inc., Chicago, IL, USA). The sociodemographic data and results of the UCSF-OCPQ were analysed using descriptive statistics. The data were tested for normal distribution with the Kolmogorov-Smirnov test, which revealed that the data do not significantly differ from normal distribution. Since the data were normally distributed, a t-test was used to compare the means of 2 interval-scaled independent samples. For categorical variables, the χ2 test was used for comparison. For all tests, P values of less than 0.05 were considered statistically significant.
